# Theranostic nanoparticles ZIF-8@ICG for pH/NIR-responsive drug-release and NIR-guided chemo-phototherapy against non-small-cell lung cancer

**DOI:** 10.1007/s10856-024-06802-1

**Published:** 2024-06-19

**Authors:** Kaiming Lu, Xiongfeng Pan, Jinyu Zheng, Dezhi Cheng, Liangcheng Zheng, Xinbo Zhang

**Affiliations:** 1 Department of Operating Room, The First Affiliated Hospital of Wenzhou Medical University, Wenzhou, 325000 PR China; 2 Department of Thoracic Surgery, The Affiliated Cangnan Hospital of Wenzhou Medical University, Wenzhou, 325800 PR China; 3 Department of Thoracic Surgery, The First Affiliated Hospital of Wenzhou Medical University, Wenzhou, 325000 PR China

## Abstract

This study leverages nanotechnology by encapsulating indocyanine green (ICG) and paclitaxel (Tax) using zeolitic imidazolate frameworks-8 (ZIF-8) as a scaffold. This study aims to investigate the chemo-photothermal therapeutic potential of ZIF-8@ICG@Tax nanoparticles (NPs) in the treatment of non-small cell lung cancer (NSCLC). An “all-in-one” theranostic ZIF-8@ICG@Tax NPs was conducted by self-assembly based on electrostatic interaction. First, the photothermal effect, stability, pH responsiveness, drug release, and blood compatibility of ZIF-8@ICG@Tax were evaluated through in vitro testing. Furthermore, the hepatic and renal toxicity of ZIF-8@ICG@Tax were assessed through in vivo testing. Additionally, the anticancer effects of these nanoparticles were investigated both in vitro and in vivo. Uniform and stable chemo-photothermal ZIF-8@ICG@Tax NPs had been successfully synthesized and had outstanding drug releasing capacities. Moreover, ZIF-8@ICG@Tax NPs showed remarkable responsiveness dependent both on pH in the tumor microenvironment and NIR irradiation, allowing for targeted drug delivery and controlled drug release. NIR irradiation can enhance the tumor cell response to ZIF-8@ICG@Tax uptake, thereby promoting the anti-tumor growth in vitro and in vivo. ZIF-8@ICG@Tax and NIR irradiation have demonstrated remarkable synergistic anti-tumor growth properties compared to their individual components. This novel theranostic chemo-photothermal NPs hold great potential as a viable treatment option for NSCLC.

**Graphical Abstract:**

**Figure:**
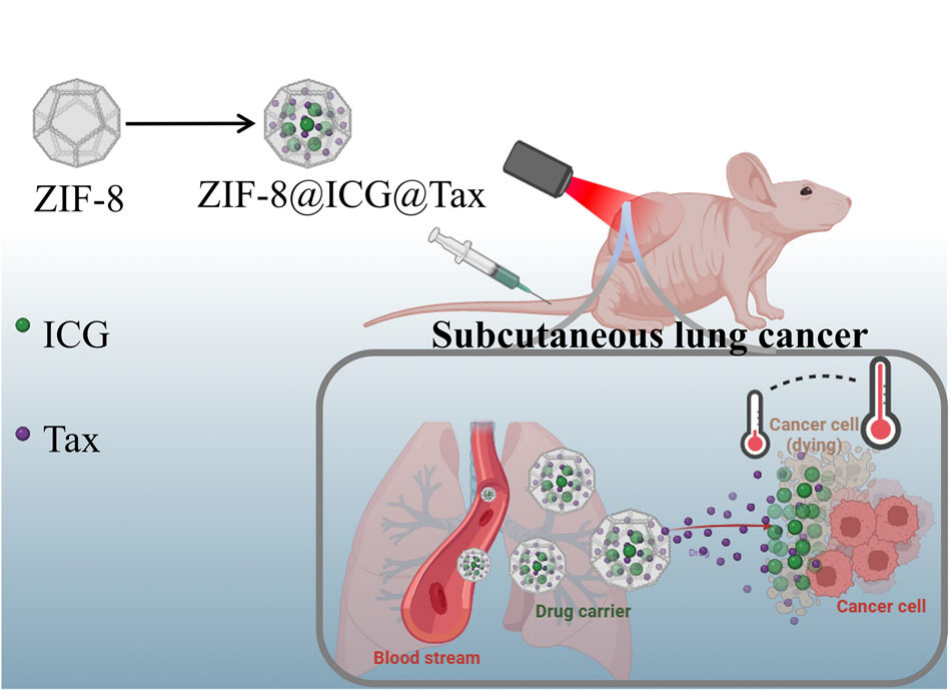
A novel nano-theranostic platform was developed by encapsulating indocyanine green and paclitaxel within a zeolitie imidazolate framework-8 (ZIF-8). This chemo-phototherapic agent demonstrated accurate tumor targeting and effective suppression effects on both tumor growth in non-small cell lung cancer.

## Introduction

Non-Small Cell Lung Cancer (NSCLC) is a prevalent form of lung cancer, accounting for ~85% of all lung cancer cases [1]. Although surgical treatment can lead to clinical cure (not complete cure) of NSCLC when performed at an appropriate time, the limitations of current clinical diagnostic techniques mean that only around 20–25% of patients are suitable for surgery upon diagnosis [2]. Due to the aggressive nature of NSCLC, the long-term survival rate of patients who undergo surgery alone is not satisfactory [1, 3]. The majority of patients must rely on antitumor drugs for treatment. However, it’s important to note that chemotherapy remains an important treatment option for advanced NSCLC, as it is an incurable disease. In the past two decades, significant advancements in treatments have been made for advanced NSCLC, including the use of novel chemotherapeutic agents such as prodrugs based on multi-functional nano-platforms. These treatments have greatly extended the overall survival of patients [4, 5].

Paclitaxel (Tax) is an anticancer diterpene alkaloid that has found extensive clinical use in the treatment of lung, breast, and ovarian cancer [6]. However, its success rate is limited, and like many conventional non-targeted chemotherapeutic agents, it is associated with severe side effects. Recognizing the inherent limitations of conventional cancer treatment, researchers have turned to nanomedicine technology to develop more effective cancer treatments [4, 7]. In recent years, the unique advantages of nanotechnology in drug delivery, diagnostics, imaging, and vaccines have attracted increasing interest from researchers seeking to improve the diagnosis and treatment of various human cancers [5].

Zeolitic imidazolate frameworks-8 (ZIF-8) with pH-responsiveness has emerged as a popular class of nanomaterials [8]. These frameworks possess excellent properties such as adjustable structure and composition, good pore size, adjustable dimensions, wide range of functionality, high drug loading capacity, and biocompatibility. As a result, they are promising carriers for drug delivery [9]. Currently, ZIF-8 has been utilized for encapsulating and delivering various components including chemotherapy drugs, photosensitizers, and proteins [10–12]. PEG-FA@ZIF-8, for instance, encapsulated with Baicalin achieved a drug loading of up to 41.45%. This system can circulate in the bloodstream under physiological conditions and gradually release Baicalin when the slightly acidic tumor microenvironment is reached in a breast cancer mouse model [13]. Combining chemotherapy with other therapeutic methods, such as phototherapy, immunotherapy, chemodynamic therapy, gene therapy, etc., has proven to be an effective approach enhance drug bioavailability, reduce side effects, and overcome tumor cell resistance, ultimately achieving a more significant therapeutic effect [14–17].

Photothermal therapy (PTT) is a non-invasive and spatiotemporally controlled therapeutic approach that utilizes photothermal agents to convert light energy into heat energy. This heat energy effectively increases the temperature of surrounding tissues, leading to the destruction of tumor cells [18]. Due to its non-invasive nature and selective targeting, PTT holds great potential in the field of oncology [19]. Indocyanine green (ICG) is an FDA-approved near-infrared (NIR) fluorescent dye with unique optical properties [20, 21]. It can be utilized not only for surgical navigation and in vivo localization diagnosis but also for converting light energy into thermal effects due to its exceptional near-infrared light absorption ability. This capability enables the induction of tumour cell stress through photothermal effects, thereby stimulating long-term anti-tumor effects [22–24]. However, it has limitations such as lack of targeting, thermal stability, and photostability [25]. To overcome these challenges, researchers developed an ICG@ZIF-8 therapeutic system that synthesized ICG@ZIF-8 nanoparticles exhibiting excellent photothermal killing abilities, ultra-high loading capacities, efficient cell uptake, excellent photothermal stability, and good biocompatibility [26].

In this study, we developed a theranostic pH- and NIR-responsive drug delivery system utilizing ZIF-8 loaded with ICG and Tax (ZIF-8@ICG@Tax) for the treatment of NSCLC. The findings indicate that NIR light can enhance the therapeutic efficacy of ZIF-8@ICG@Tax by promoting its uptake by tumor cells. Moreover, ZIF-8@ICG@Tax effectively mitigates the toxic side effects associated with conventional chemotherapeutic drugs. This combination approach demonstrated enhanced anti-tumor and anti-metastatic effects. These results are encouraging and suggest promising future applications for such integrated approaches.

## Materials and methods

### Preparation of ZIF-8@ICG@Tax

The preparation of ZIF-8@ICG@Tax nanoparticles (NPs) involved dissolving 5 mg of ICG (abs816408, absin, Shanghai, China) and 10 mg of Tax (S1150, Selleck, Texas, USA) in 100 μL of dimethyl sulfoxide (DMSO, ST038, Beyotime, Shanghai, China) solvent. Additionally, 330 mg of 2-methylimidazole (693-98-1, Meilunbio, Shanghai, China) and 150 mg of zinc nitrate hexahydrate (10196-18-6, Yihe Biological, Shanghai, China) were dissolved in 5 mL of ultrapure water. Next, at a temperature of 20 °C, 5 mL of ultrapure water was gradually added into a solution containing 10 mL of methanol while continuously stirring for 20 min. The resulting mixture was then centrifuged at 12,000 rpm for 15 min to remove any incomplete reagents. To further refine the ZIF-8@ICG@Tax NPs, they were subjected to methanol centrifugation at 12,000 rpm for 15 min, repeating the process three times.

### Characterization of ZIF-8@ICG@Tax

The morphology, microstructure, and composition of ZIF-8@ICG@Tax NPs were characterized using transmission electron microscopy with the FEI Talos F200X instrument from the USA. The crystalline structure of ZIF-8 and ZIF-8@ICG@Tax were investigated using X-ray diffraction (XRD) with the Smart Lab instrument from Japan. Fourier infrared analysis was conducted using the Tensor II instrument from Germany. Furthermore, the particle size and zeta potential of ZIF-8 and ZIF-8@ICG@Tax were determined using Zetasizer Nano.

### In vitro ZIF-8@ICG@Tax photothermal performance

The photothermal conversion efficiency of the materials was evaluated using a thermal imager. ZIF-8, ICG, Tax, and ZIF-8@ICG@Tax were prepared with water at a concentration of 50 μg/mL. Then, 200 μL of the solution was pipetted into a 1.5 mL centrifuge tube. The sample was subjected to an 808 nm laser beam for 10 min. A thermal imager (FOTRIC 220 s, Feiquco Intelligent Technology, China) was employed to perform NIR irradiation. The temperature value was recorded every 30 s, and an infrared thermogram image was captured every 1 min. Furthermore, to investigate the effect of concentration and optical density on temperature values, different concentrations of ZIF-8@ICG@Tax were prepared, and the current level of the laser beam was adjusted accordingly, as described above. Temperature values and infrared thermograms corresponding to various concentrations and optical densities were obtained.

To assess the photothermal stability, the laser beam was turned on and off five times consecutively, and the corresponding temperature values were recorded. The photothermal conversion efficiency (*η*) was calculated using the following equation [27]:

$$\eta =\frac{{hs}({{\rm{T}}}_{\max }-{{\rm{T}}}_{{\rm{S}}})-{Q}_{{Dis}}}{I(1-{10}^{-A})}$$

Among these variables, “*h*” represents the thermal conversion coefficient, while “s” denotes the surface area of the container. “Tmax” signifies the highest temperature achieved, “*T*s” represents the room temperature, “*I*” stands for laser power, “*A*” symbolizes the absorbance of the dispersion at 808 nm, and “QDis” represents the heat absorbed and dissipated by the solvent and container.

### In vitro drug-releasing calculations

To prepare ZIF-8@ICG@Tax for pH-responsive releasing testing, it was adjusted to a concentration of 1 mg/mL using PBS. The solution was shaken and stirred at pH 5.5 and 7.4 for various time points: 0, 1, 2, 4, 6, 8, 12, 18, 24, 36, and 48 h. At each time point, the samples were isolated by filtration through a membrane.

To prepare ZIF-8@ICG@Tax for NIR-responsive releasing testing, it was adjusted to a concentration of 50 μg/mL using PBS. The cells were conducted in two groups: the group without NIR irradiation (-NIR group) and the group with NIR irradiation (+NIR group). In the +NIR group, a 1.5 mL tube containing 200 μL of ZIF-8@ICG@Tax solution was irradiated with a laser at 808 nm and a power density of 1.0 W cm^-2^ for 10 min. After irradiation, the solution was collected by filtration through a membrane at various time points including 0, 1, 2, 4, 6, 8, 12, 18, 24, 36, and 48 h. Subsequently, the isolate was mixed with 10 mL of PBS. Similarly, in the -NIR group, the solution was filtered through a membrane at the same time points to obtain the isolate without NIR irradiation.

Finally, the concentration of Tax in the isolates was determined using High-Performance Liquid Chromatography, and the drug release rate was calculated following:

$${Release\; rate}=\frac{{\rm{m}}({\rm{Releasing}})}{{\rm{m}}({\rm{Loading}})}\times 100 \%$$

### Cell culture

HLF and A549 cells were obtained from Heifei Wanwu Biological Technology Co., Ltd. HLF cells were cultured in RPMI-1640 DMEM (Invitrogen) containing 10% FBS. A549 cells were cultured in F12K DMEM (Invitrogen) containing 10% FBS (Gibco). All cells were maintained under 37 °C and 5% CO_2_ conditions.

### Cell counting kit-8 (CCK-8)

The cells from each treatment group were converted into a single-cell suspension, and their count was determined. Subsequently, the cells were seeded in 96-well plates at a density of 5000 cells per well with 100 μL of culture medium in each well. Each concentration/treatment group consisted of six replicates.

To determine the half-maximal inhibitory concentration (IC50), a gradient concentration of ZIF-8@ICG@Tax was added to the culture medium in the wells. In A549 cells, various concentrations of ZIF-8@ICG@Tax containing 1, 2, 4, 8, 16, 20, 30, 40 µg/mL were applied, where in HLF cells, the concentrations applied were 5, 10, 20, 40, 80, 100, 150, 200, and 250 µg/mL. After treating cells with the gradient concentration of ZIF-8@ICG@Tax for 24 h, they were subjected to NIR irradiation (808 nm, 1.0 W/cm^−2^) for 10 min.

To access cell viability following different treatments, A549 cells were exposed to PBS, Tax (5 µg/mL) and ZIF-8@ICG@Tax (25 µg/mL) for 4 h. Then, the cells were cultured for an additional 10 min, with or without NIR (808 nm, 1.0 W/cm^-2^).

For the CCK-8 incubation, 10 μL of CCK-8 solution was added to each well. The plates were then returned to the incubator and incubated for an additional 2 h. After incubation, the absorbance at 450 nm was measured using a spectrophotometer.

### LIVE/DEAD staining

Staining was performed using the Calcein-AM/PI Live and Dead Staining Kit (Bebo, Shanghai, China). In brief, cells were divided into five groups and seeded into 48-well cell culture plates. Each well was inoculated with 1 × 10^4^ cells, and three replicate wells were established for each group. After 24 h of incubation, the α-MEM medium was replaced with α-MEM medium containing PBS, Tax (5 µg/mL) and ZIF-8@ICG@Tax (25 µg/mL) for 4 h. The cells were then cultured for an additional 10 min, with or without NIR (808 nm, 1.0 W/cm^−2^). Following the incubation period, the cells were treated with appropriate concentrations of Staining A and B liquids from the Calcein-AM/PI Live and Dead Staining Kit. Subsequently, the cells were incubated in darkness for a specific duration. After the incubation, the cells were washed three times with PBS and observed under a fluorescence microscope.

### Flow cytometry

Apoptosis was assessed using flow cytometry. A549 cells were seeded in 6-well plates and cultured for 12 h. Subsequently, the cells were treated with PBS, Tax (5 µg/mL) and ZIF-8@ICG@Tax (25 µg/mL) for 4 h. Then, the cells were subsequently incubated for another 10 min, with or without NIR (808 nm, 1.0 W/cm^−2^). After the incubation period, the cells were collected and apoptosis was detected using the Annexin V-FITC/PI apoptosis kit (Multi Sciences, Hangzhou, China) according to the provided instructions. The stained cells were promptly analyzed on a flow cytometry machine upon completion of the staining process.

### Cellular uptake experiments

The cellular uptake of ZIF-8@ICG@Tax in A549 cells was evaluated using confocal laser scanning microscopy (CLSM). A549 cells were seeded at a concentration of 1 × 10^5^ cells/mL on 35 mm glass-bottomed petri dishes and incubated overnight. Following the incubation, the cells were treated with serum-free DMEM medium containing ZIF-8@ICG@Tax at a concentration of 25 µg/mL for duration of 4 h. Subsequently, the cells were then cultured for an additional 10 min, with the option of NIR (808 nm, 1.0 W/cm^−2^) or without it. After the incubation period, the cells were washed three times with PBS and fixed with 4% paraformaldehyde for 15 min. Subsequently, images of the cells were captured using confocal laser scanning microscopy to visualize and analyze the uptake of ZIF-8@ICG@Tax.

### Hemolysis assay

1 mL of fresh anticoagulation-treated mouse blood was obtained. To prepare the samples, the blood was mixed with ten times the volume of PBS solution in a centrifuge tube. The mixture was then centrifuged at a rate of 2000 rpm for 10 min. After removing the supernatant, the mixture was diluted with PBS to create a 2% erythrocyte suspension. Next, a gradient concentration of ZIF-8@ICG@Tax solution and added it to a centrifuge tube containing 0.6 mL of the erythrocyte suspension. This resulted in final concentrations of 0, 12.5, 25, 50, 100 and 200 μg/mL, with a total volume of 1.5 mL for each sample.

Subsequently, all the samples, including the experimental groups and controls, were incubated in a 37 °C water bath for 1 h. After incubation, the samples were centrifuged at a rate of 3000 rpm for 15 min. Following centrifugation, photographs were taken, and the absorbance of each sample was measured at 570 nm using a multifunctional enzyme marker.

Finally, we calculated the hemolysis rate using the following formula [28]:

$${Hemolytic\; Ratio}\left( \% \right)=\frac{{OD}\left({sample}\right)-{OD}\left({negative}\right)}{{OD}\left({positive}\right)-{OD}\left({negative}\right)}\times 100 \%$$

OD (sample), OD (negative), and OD (positive) represent the absorbance of the sample, negative control, and positive control, respectively.

### Animal model and treatment

The animal experimental procedures described in the study were conducted in compliance with the regulations and guidelines set by the Animal Ethics Committee. Fifty Balb/c-Nude male mice (6 weeks old) were procured from Sparfo Technology for various in vivo experiments including treatments and blood biochemistry analyses. The treatment time points for tumor-bearing mice vary across different models. However, in all cases, the amount of Tax and ZIF-8@ICG@Tax injected were consistent at 2 mg/kg, and the PBS group received the same volume of PBS.

Twenty-five Balb/c-Nude female mice were randomly divided into five groups: (1) PBS, (2) PBS + NIR, (3) Tax, (4) ZIF-8@ICG@Tax, and (5) ZIF-8@ICG@Tax+NIR, with five mice in each group. In the Tax group, a tail vein injection of 100 µL of Tax at a dose of 2 mg/kg was administered. In the ZIF-8@ICG@Tax group and ZIF-8@ICG@Tax+NIR group, a tail vein injection of 100 µL of ZIF-8@ICG@Tax at a dose of 2 mg/kg was administered. The control group received a tail vein injection of an equal volume of PBS. After the completion of treatment, blood samples were collected through the eyeballs for blood routine and liver and kidney function index testing.

Subcutaneous tumor transplantation model in mice: The mice should be placed under anesthesia on a sterile workbench. To determine the position of the lungs, make a small incision of ~5 mm on the epidermis and gradually dissect the underlying subcutaneous and muscle tissue. Then, inject 200 µL of a mixed solution containing 5 × 10^5^ A549 cell suspension slowly along the incision towards the left lung of the mouse, ensuring a depth of approximately 3 mm. After injection, pause for 5 s, gently rotate the needle left and right, and then withdraw it before suturing the wound. The tumors were allowed to grow to approximately 100 mm^3^ before being divided into five groups: (1) PBS, (2) PBS + NIR, (3) Tax, (4) ZIF-8@ICG@Tax, and (5) ZIF-8@ICG@Tax+NIR, with five mice in each group. On the first day after grouping, the Tax group received a subcutaneous injection of 100 µL of Tax at a dose of 2 mg/kg, while the ZIF-8@ICG@Tax group and ZIF-8@ICG@Tax+NIR group received a subcutaneous injection of 100 µL of ZIF-8@ICG@Tax at a dose of 2 mg/kg. The control group received a subcutaneous injection of an equal volume of PBS. Subsequently, the mice were exposed to a laser beam (808 nm, 1.0 W/cm^−2^) for 10 min. Temperature values were recorded every 1 min using a thermal imager (FOTRIC 220s, Fei Zhi Ke Intelligent Technology, Shanghai, China), and an infrared imaging picture was taken. After continuing to observe the mice for 13 days, the mice were euthanized, and tumor tissue samples were collected for HE staining.

### HE staining

Paraffin sections underwent a series of steps for processing and staining. Firstly, the sections were dewaxed using xylene and rehydrated with graded concentrations of alcohol. Subsequently, they were washed with PBS and immersed in a hematoxylin staining solution for 10 min to enable visualization of cell nuclei. Following tap water immersion, the slides were briefly exposed to dilute hydrochloric acid alcohol for 5 s, then treated with light ammonia for 5 min. Next, eosin staining was performed by immersing the slides in eosin solution for 10 min. To ensure proper dehydration, the slides were sequentially immersed in 70%, 80%, and 90% alcohol for 1 min each, subsequent to tap water immersion. They were then subjected to two 1-min immersions in 95% alcohol, followed by three 1-min immersions in anhydrous ethanol using xylene as a medium. Finally, the slides were sealed with neutral resin. After washing the slides with tap water, a dehydration process was performed by immersing them successively in 70%, 80%, and 90% alcohol for 1 min each. This was followed by immersions in 95% alcohol, anhydrous ethanol, and xylene, each for 1 min. Ultimately, the slides were sealed with neutral resin.

### Data analysis

In this study, we conducted statistical analysis on the results of at least three independent experiments. The experimental data were presented as mean ± standard deviation (SD) and analyzed using GraphPad Prism 8.0 software. Statistical analysis was performed using one-way ANOVA. A *p*-value of less than 0.05 was considered statistically significant, indicating a significant difference between the results.

## Results

### Characterization of ZIF-8@ICG@Tax

**Fig. 1:**
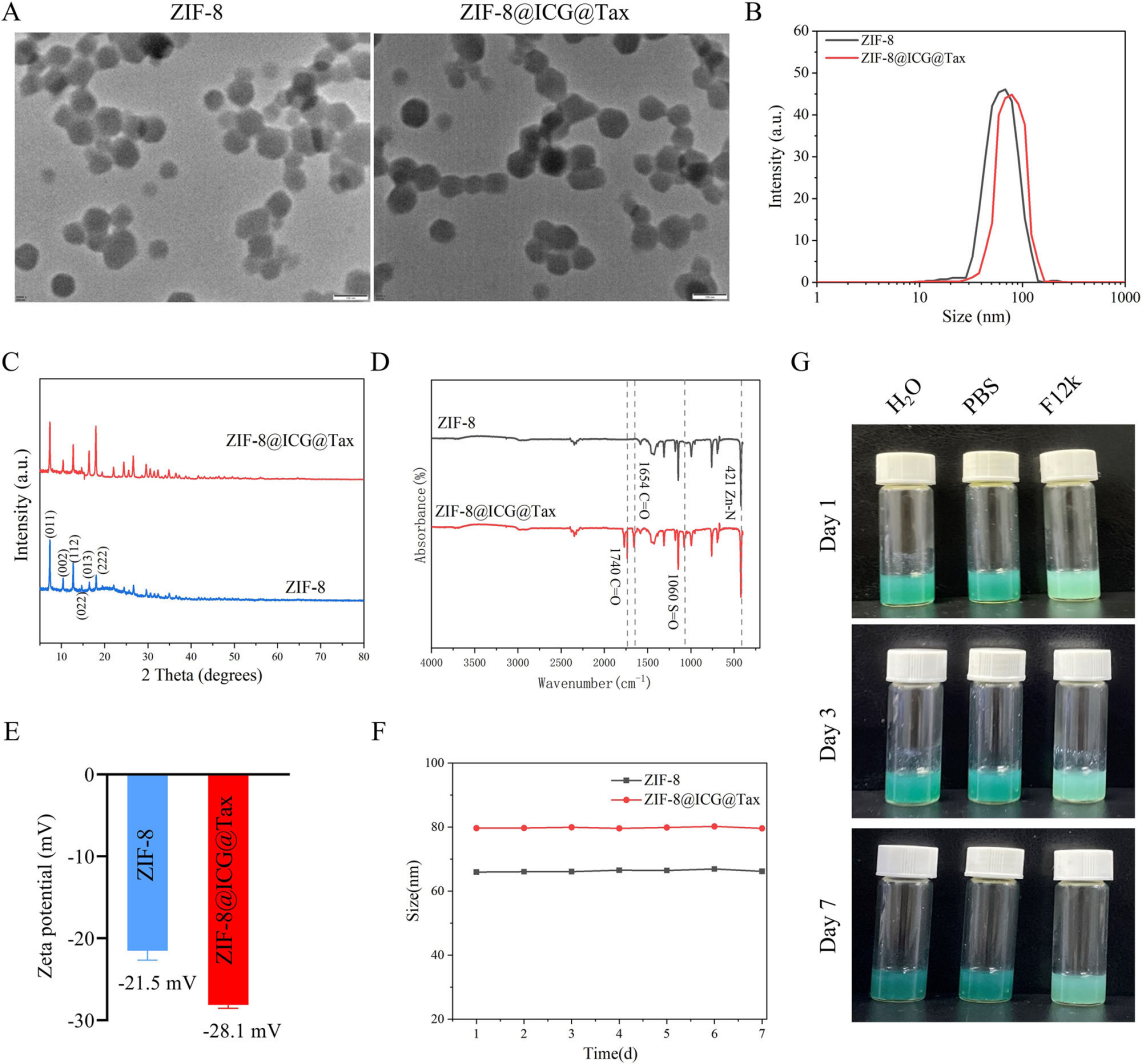
Characterization of ZIF-8@ICG@Tax. **A** Transmission electron microscopy (TEM) to detect the surface morphology and internal structure of ZIF-8 and ZIF-8@ICG@Tax; **B** dynamic light scattering (DLS) to detect the average size distribution of ZIF-8 and ZIF-8@ICG@Tax; **C** X-ray diffraction (XRD) detection of the crystalline structure of ZIF-8 and ZIF-8@ICG@Tax; **D** Fourier transform infrared spectroscopy (FTIR) to identify the molecular structure of ZIF-8@ICG@Tax Particle; **E** Zeta potential measurement of ZIF-8 and ZIF-8@ICG @Tax zeta potential measurements; **F**, **G** stability of ZIF-8 and ZIF-8@ICG@Tax in different solvents

We initially investigated the characterization of ZIF-8 and ZIF-8@ICG@Tax NPs. It was observed that ZIF-8 exhibited a homogeneous and typical polyhedral morphology, while ZIF-8@ICG@Tax did not show any significant change in morphology after loading with ICG and Tax (Fig. 1A). This suggests that the loading of ICG and Tax does not affect the basic structure and morphology of ZIF-8.

The size of NPs plays critical role in their cellular uptake efficiency. The optimal particle size for cell uptake is generally considered to be less than 100 nm [29]. The average particle sizes of ZIF-8 and ZIF-8@ICG@Tax were approximately 65.88 ± 0.11 nm and 77.13 ± 2.75 nm, respectively (Fig. 1B). Therefore, both ZIF-8 and ZIF-8@ICG@Tax nanoparticles were within the suitable range for efficient cellular uptake. Moreover, the larger particle size of ZIF-8@ICG@Tax compared to that of ZIF-8 suggested that ICG and Tax have been successfully loaded onto the ZIF-8 framework.

Crystal structure analysis showed that the XRD peaks of ZIF-8@ICG@Tax were almost identical to those of ZIF-8, indicating that the loading of ICG and Tax did not affect the crystal structure of ZIF-8 (Fig. 1C). Moreover, the crystallinity of the samples closely matched the reported crystal structure data of ZIF-8, confirming the successful synthesis of ZIF-8 [30].

Additionally, the analysis of absorption peaks revealed that the peak at 421 cm^−1^ corresponded to the stretching vibration of the zinc-nitrogen bond in ZIF-8. Moreover, peaks at 1740 cm^−1^ and 1654 cm^−1^ indicated the presence of carbonyl and aromatic ketone of paclitaxel, respectively, while the peak at 1060 cm^−1^ corresponded to the S=O stretching vibration of ICG (Fig. 1D). Moreover, the zeta potential of ZIF-8@ICG@Tax was lower compared to that of ZIF-8, indicating that ICG and Tax were anchored to ZIF-8 in the form of negative charges (Fig. 1E). These results indicate that we successfully loaded PTX and ICG onto ZIF-8 NPs.

Additionally, ZIF-8@ICG@Tax exhibited good dispersion and stability in water, PBS, and cell culture medium, maintaining this stability for at least 7 days (Fig. 1F, G). The ability of ZIF-8@ICG@Tax to maintain its stability over time in different environments suggests that it could potentially be a promising drug delivery system [31].

In summary, these findings confirm the successful synthesis of ZIF-8 and ZIF-8@ICG@Tax NPs with homogeneous morphology and fine stability.

### In vitro photothermal effect, stability and drug release of ZIF-8@ICG@Tax

**Fig. 2:**
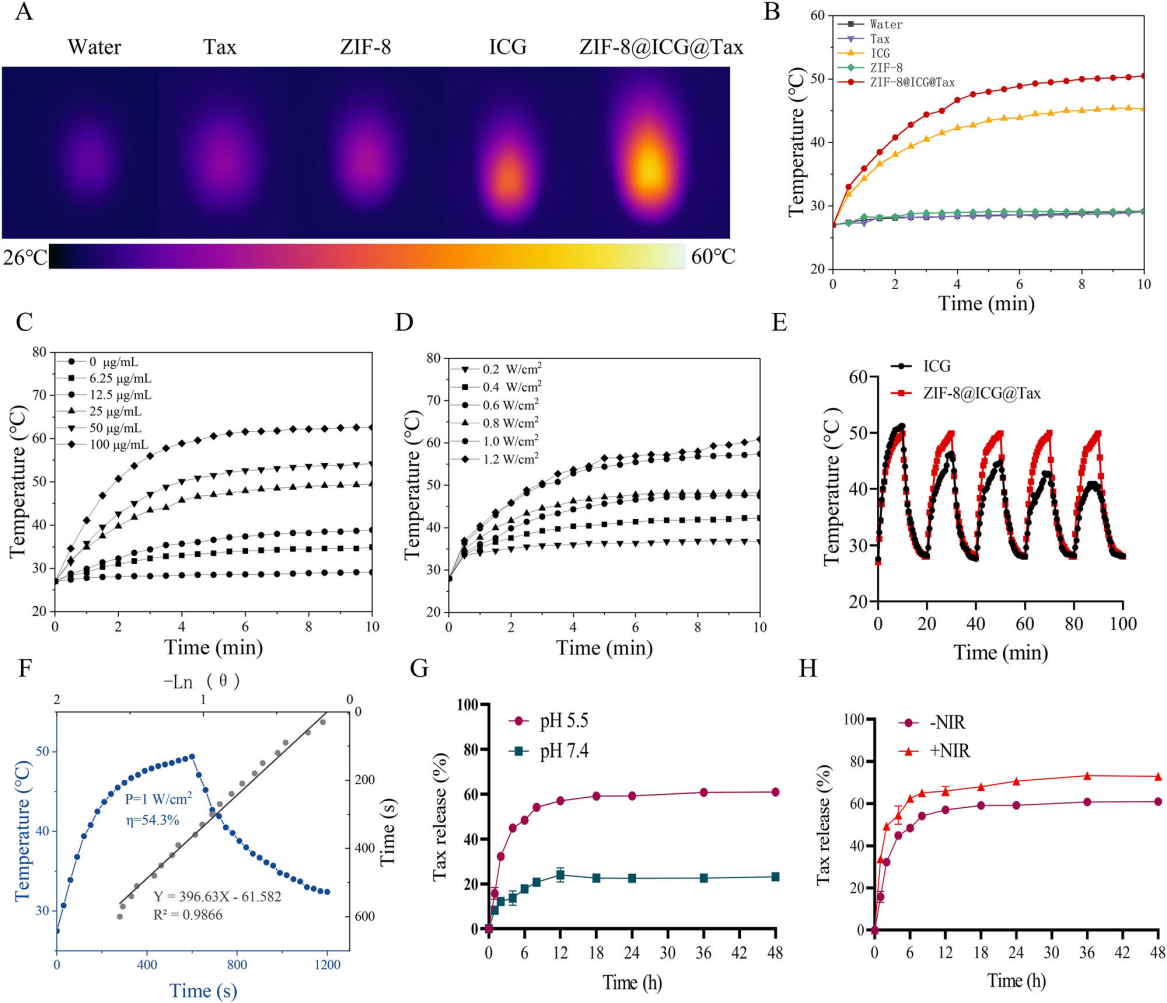
In vitro photothermal effect, stability and drug release of ZIF-8@ICG@Tax. **A**, **B** NIR laser irradiation to detect the photothermal properties of ZIF-8@ICG@Tax; **C** 808 nm IR laser irradiation of different concentrations of ZIF-8@ICG@Tax to detect the photothermal effect versus concentration; **D** irradiation of 50 μg/mL of ZIF-8@ICG@Tax with different laser densities to detect the photothermal effect versus optical density; **E** Temperature changes of ICG and ZIF-8@ICG@Tax during five switching cycles; **F** The photothermal conversion efficiency of ZIF-8@ICG@Tax; **G**, **H** Tax release from ZIF-8@ICG@Tax in different pH environments and with or without NIR irradiation

The in vitro photothermal conversion properties of ZIF-8@ICG@Tax were evaluated using a NIR irradiation (808 nm, 1.0 W cm^-2^) on solutions containing water, ICG (11 μg/mL), Tax (5 μg/mL), ZIF-8 (25 μg/mL), and ZIF-8@ICG@Tax (25 μg/mL) for a duration of 10 min. The temperature changes of those different solutions were monitored using IR thermal images. The results demonstrated that upon NIR irradiation, both ICG and ZIF-8@ICG@Tax experienced an increase in temperature. Notably, the temperature of ZIF-8@ICG@Tax exhibited the largest increase, rapidly rising from 27 °C to 54.2 °C (Fig. 2A, B). Moreover, we observed a correlation between the temperature elevation of ZIF-8@ICG@Tax under 808 nm irradiation and the drug concentration as well as the optical density (Fig. 2C, D). These findings suggest that ICG-induced PTT can efficiently and rapidly convert light energy into thermal energy when exposed to an 808 nm laser. Consequently, this synthesized nano-drug ZIF-8@ICG@Tax exhibits promising potential for PTT.

The in vitro photothermal stability of ZIF-8@ICG@Tax was evaluated, with free ICG as a control. Remarkably, compared to free ICG, ZIF-8@ICG@Tax maintained stable photothermal conversion efficiency throughout the cycles (Fig. 2E). This outstanding stability can be attributed to the synergistic coordination interactions that provide enhanced stability, effectively addressing the issue of ICG’s easy photobleaching tendency [32].

Based on calculations, the photothermal conversion efficiency (*η*) of HAVH was determined to be 54.3% (Fig. 2F), which indicates its superior conversion ability compared to other reported photothermal agents, such as HCuS carrier with an efficiency of 38.4% [33]. Taken together, it can be concluded that ZIF-8@ICG@Tax possesses reliable photothermal and photodynamic properties.

The design of pH-responsive nanomedicine delivery carriers plays a crucial role in harnessing the pH disparity between tumor and normal tissues. These carriers are responsible for maintaining drug stability under physiological conditions while facilitating targeted aggregation and release at the tumor site [34, 35]. Therefore, we examined the release of ZIF-8@ICG@Tax under different pH and irradiation conditions. Compared to the conditions of pH 7.4 and no-NIR irradiation, both pH 5.5 and the presence of NIR irradiation significantly enhance the rate of drug release. In particular, the addition of NIR irradiation can achieve a release rate of 66% for Tax within 12 h (Fig. 2G, H), highlighting the potential and application of ZIF-8@ICG@Tax as an effective drug carrier.

### In vitro antitumor effect of ZIF-8@ICG@Tax

**Fig. 3:**
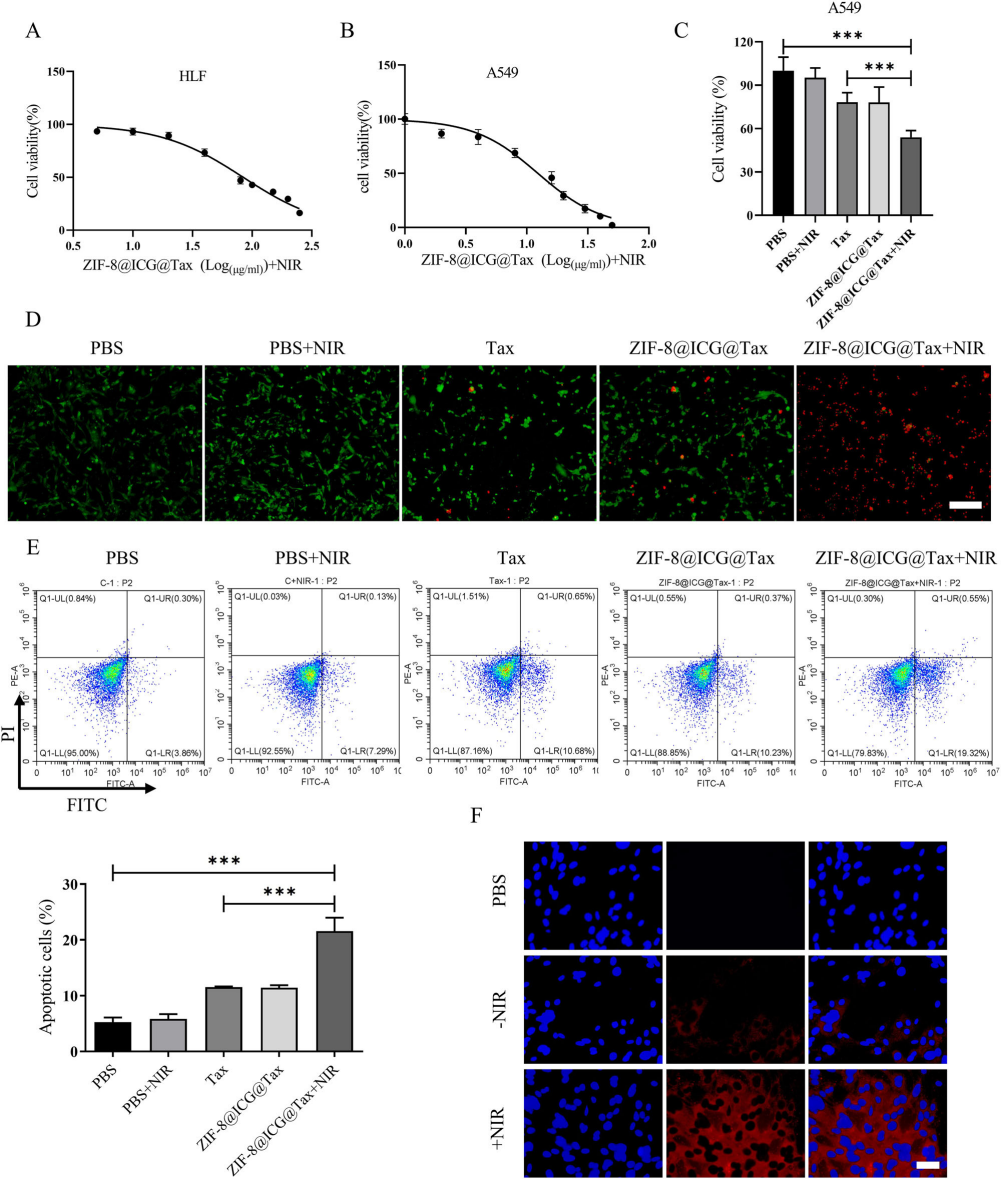
In vitro anti-tumor effect of ZIF-8@ICG@Tax. **A**, **B** CCK-8 to detect the killing effect of different concentrations of ZIF-8@ICG@Tax on lung fibroblast-like cell line HLF and human NSCLC cell line A549; **C**–**E** CCK-8, PI/Calcein-AM dual fluorescence live-dead staining and flow cytometry to detect the killing effect of different treatments on A549 cells. Scale bar: 200 µm; **F** DAPI staining to detect the effect of NIR on cellular uptake of ZIF-8@ICG@Tax. Scale bar: 50 µm. *** *P* < 0.001

We initially treated the lung fibroblast-like cell HLF and NSCLC cell line A549 with ZIF-8@ICG@Tax (Fig. 3A, B). We found that ZIF-8@ICG@Tax significantly induced apoptosis in A549 cells (IC50 = 12.33 μg/mL, 24 h), while exhibiting a lesser effect on HLF cells (IC50 = 84.21 μg/mL, 24 h). Based on the IC50 value, we selected a concentration of 25 μg/mL of ZIF-8@ICG@Tax for the subsequent in vitro experiments. When ZIF-8@ICG@Tax was irradiated with an 808 nm laser, it demonstrated the highest therapeutic effect in vitro by effectively inhibiting the activity of A549 cells (Fig. 3C–F). This indicates the excellent PTT ability of chemo-photothermal ZIF-8@ICG@Tax NPs. Notably, the combined use of ZIF-8@ICG@Tax NPs and NIR irradiation demonstrates superior tumor-killing efficacy when compared to free Tax alone. This enhanced therapeutic effect can be attributed to the specific accumulation of ZIF-8@ICG@Tax at the tumor site, resulting in improved anti-tumor activity against A549 cells. These findings highlight the promise of this combined approach for targeted drug delivery and cancer treatment.

The efficiency of cell uptake is important in the effectiveness of nano-mediated cancer treatment. ICG, due to its binding effect with intracellular glutathione S-transferase, was predominantly distributed in the cytoplasm (Fig. 3G). Interestingly, in the group subjected to NIR laser irradiation, the ICG signal within A549 cells significantly increased (Fig. 3G). This phenomenon can be attributed to the generation of high heat caused by the laser, as well as the nanomaterial’s promotion of cellular uptake [36, 37].

Taken together, our findings suggest that the application of NIR irradiation can enhance the cellular uptake efficiency of ZIF-8@ICG@Tax and augment its anti-tumor effects in cancer cells.

### Biocompatibility evaluation of ZIF-8@ICG@Tax

**Fig. 4:**
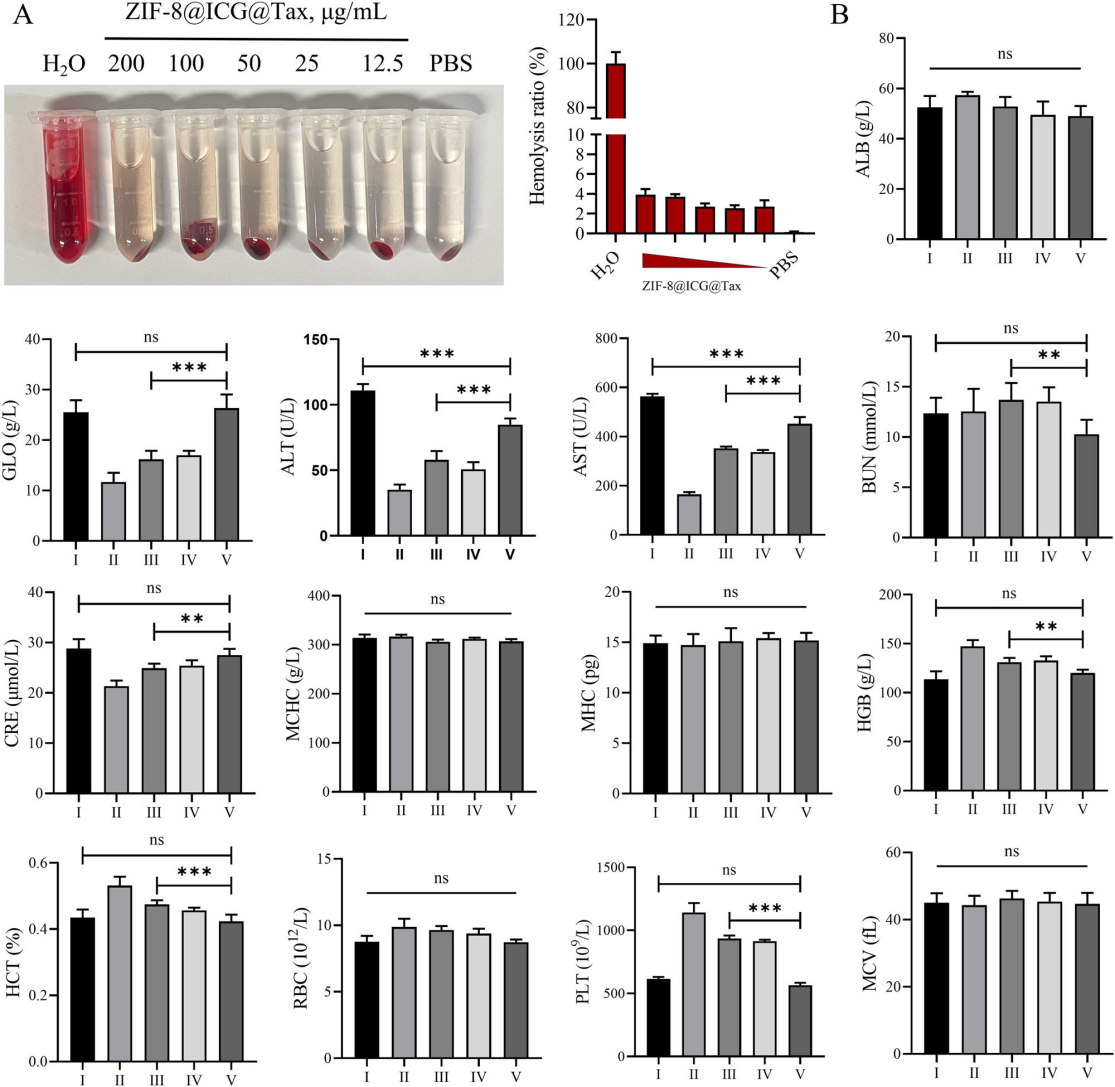
Biocompatibility evaluation of ZIF-8@ICG@Tax. **A** Hemolysis assay to assess the blood compatibility of ZIF-8@ICG@Tax; **B** Biochemical assay to detect the changes of liver and kidney function-related indexes in serum of mice; I: PBS; II: PBS + NIR; III: Tax; IV: ZIF-8@ICG@Tax; V: ZIF-8@ICG@Tax+NIR

In light of the efficient targeting potential of ZIF-8@ICG@Tax towards cancer cells and its remarkable anti-proliferation and pro-apoptosis activity, we investigated the feasibility of in vivo application of ZIF-8@ICG@Tax. The in vitro biosafety of ZIF-8@ICG@Tax was assessed using a hemolysis assay, which demonstrated that even at a concentration of 200 μg/mL, the hemolysis rate did not exceed 5% (Fig. 4A). This indicates that ZIF-8@ICG@Tax exhibits excellent hemocompatibility and holds promise for intravenous therapy.

Considering the direct relationship between in vivo toxicity of antitumor drugs and patient survival and medication adherence [38], we evaluated the effects of ZIF-8@ICG@Tax+NIR in mice. Mice were injected with 100 μL of PBS, Tax, and ZIF-8@ICG@Tax via the tail vein, and their physiological toxicity was subsequently monitored. Hepatorenal toxicity was assessed by measuring ALB, GLO, ALT, BUR, CRE, MCHC, MHC, HGB, HCT, RBC, PLT, and MCV in the serum of mice. Compared to the PBS group, treatment with NIR, Tax, and ZIF-8@ICG@Tax significantly decreased the levels of GLO, ALT, AST, and GRE in mice, while increasing the levels of BUR, HGB, and HCT. Notably, the combination of ZIF-8@ICG@Tax+NIR showed significant mitigation of the changes in the aforementioned indicators (Fig. 4B). These findings suggest that ZIF-8@ICG@Tax+NIR can effectively reduce the hepatorenal toxicity associated with Tax treatment.

Collectively, these findings indicate that ZIF-8@ICG@Tax+NIR treatment is biocompatible and could reduce both liver and kidney toxicity in mice. These results further substantiate the safety and feasibility of utilizing ZIF-8@ICG@Tax as a potential antitumor therapeutic agent.

### In vivo antitumor effect of ZIF-8@ICG@Tax

**Fig. 5:**
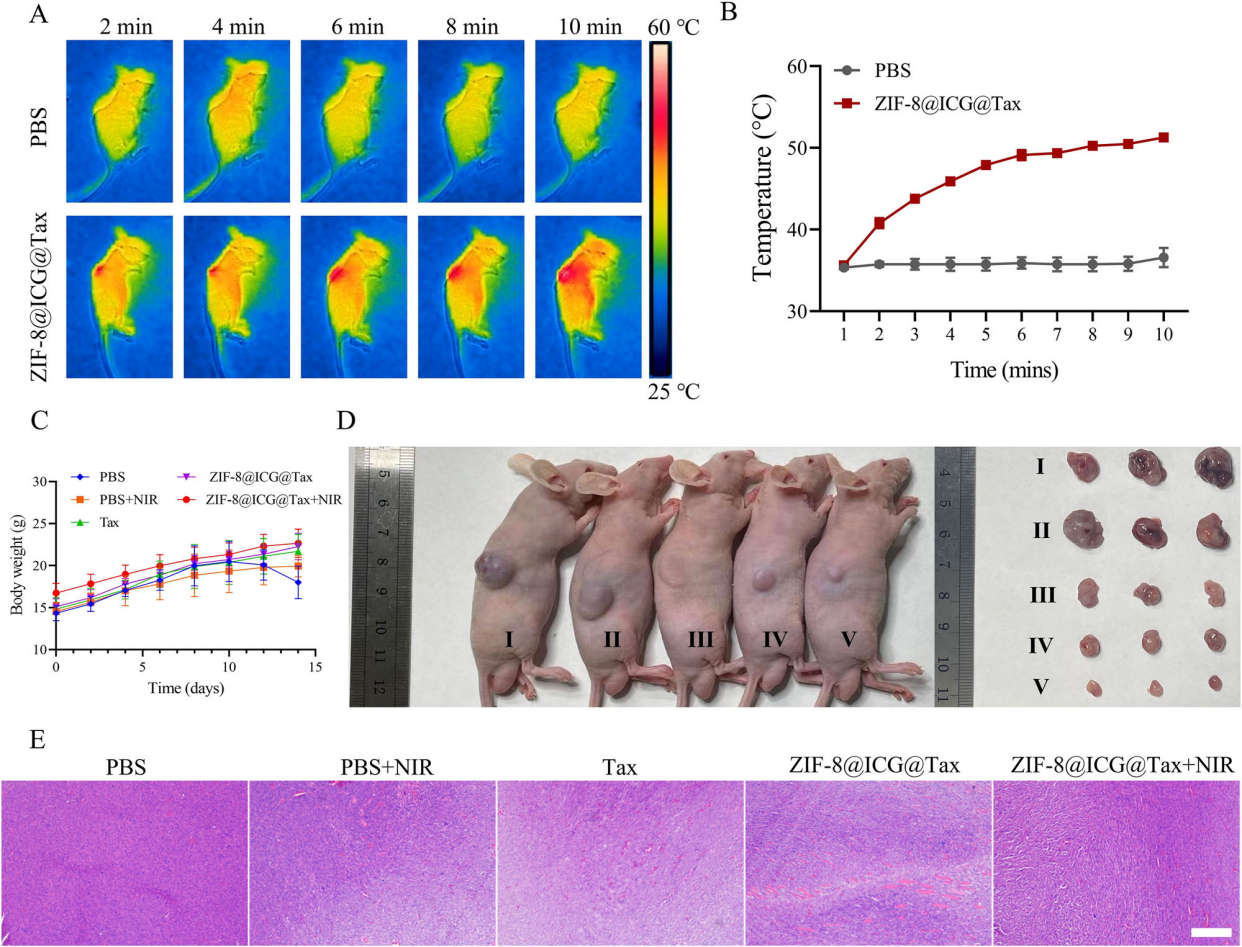
In vivo anti-tumor effect of ZIF-8@ICG@Tax. **A**, **B** Photothermal properties of ZIF-8@ICG@Tax detected by in vivo imaging in mice; **C** Body weight of mice in each group; **D** Tumorigenic volume of mice in each group; **E** HE staining to detect pathological changes of tumorigenicity in mice in each group. Scale bar: 200 µm. I: PBS; II: PBS + NIR; III: Tax; IV: ZIF-8@ICG@Tax; V: ZIF-8@ICG@Tax+NIR

Based on the favorable blood compatibility of the nanomaterials, we conducted further investigations into their in vivo applications. To confirm the synergistic chemo-phototherapeutic effects of ZIF-8@ICG@Tax, we assessed its antitumor efficacy in tumor-bearing mice. We initially verified the in vivo photothermal performance of ZIF-8@ICG@Tax by irradiating the tumor site of mice for 10 min, resulting in a temperature increase from 35.6 °C to 51.3 °C (Fig. 5A, B). These results suggested ZIF-8@ICG@Tax exhibit outstanding potential for PTT in vivo. Meanwhile, the mice in the PBS and PBS + NIR treatment groups exhibited minor weight loss (Fig. 5C).

As previously reported, the administration of free drug (Tax) alone has shown limited in vivo metabolic characteristics, leading to inadequate inhibition of tumor growth [39]. However, when chemotherapy and PTT were combined, the use of ZIF-8@ICG@Tax exhibited improved chemotherapy effects compared to the groups treated with PBS or free drug alone. Moreover, building upon our in vitro findings, the combination therapy involving ZIF-8@ICG@Tax and NIR irradiation demonstrated the most pronounced reduction in tumor burden. This can be attributed to the targeted aggregation of ZIF-8@ICG@Tax in tumor cells, resulting in enhanced therapeutic efficacy (Fig. 5D). The antitumor effect of ZIF-8@ICG@Tax was further substantiated through histological examination using HE staining. Notably, the combination of NIR irradiation and ZIF-8@ICG@Tax demonstrated a remarkable outcome with severe necrosis observed in the tumor tissue (Fig. 5E). These results highlight the potential of ZIF-8@ICG@Tax-based chemo-phototherapy as a promising approach for effective treatment of NSCLC.

Taken together, these findings suggest that ZIF-8@ICG@Tax demonstrates its most potent antitumor effect in mice through the synergistic combination of PTT and chemotherapy, along with its targeted effect on tumor cells.

## Discussion

The targeted delivery of chemotherapy drugs using biomaterials has been a hot topic in cancer research. In recent years, many new methods, such as photothermal therapy, have shown significant therapeutic effects on tumors while exhibiting low immunogenicity [26]. Therefore, we speculate that utilizing biomaterials for targeted delivery of chemotherapy drugs combined with photothermal therapy could be a promising anti-tumor treatment. Based on this, we synthesized pH-responsive ZIF-8@ICG@Tax biomaterial, which demonstrated good biocompatibility and photothermal conversion efficiency, and exhibited superior anti-NSCLC efficacy both in vitro and in vivo.

It is well known that the extracellular environment of tumor tissues is mostly acidic [40]. Therefore, we chose pH-responsive nanomaterial ZIF-8, which is easily synthesized, functionalized, has high drug-loading capacity, and rapidly degrades in acidic conditions, releasing Tax to achieve targeted therapy for NSCLC [13]. Additionally, we introduced NIR fluorescent dye ICG into the material. Although ICG has good photothermal properties, it lacks targeting ability and photothermal stability [25]. According to our results, ZIF-8@ICG@Tax exhibited significantly improved photothermal performance compared to ICG alone, thanks to the complementary properties of ZIF-8. It has been reported that ZIF-8 can effectively load ICG and enhance its stability [26]. The photothermal conversion efficiency (η) of our synthesized ZIF-8@ICG@Tax reached 54.3%, which is comparable to or even better than many previously reported photothermal nanomaterials [41–43].

In this study, ZIF-8@ICG@Tax demonstrated stronger anti-NSCLC effects both in vitro and in vivo compared to free Tax. The reasons behind this observation can be attributed to two factors. Firstly, pH-responsive ZIF-8 exhibited targeted accumulation in tumors, thereby enhancing the utilization of Tax. Secondly, there was a synergistic effect between chemotherapy and photothermal therapy. The advantages of this combined treatment strategy based on biomaterial-mediated targeted delivery of chemotherapy drugs and phototherapy include the use of lower doses of chemotherapy drugs, elimination of adverse cytotoxic effects, and improved therapeutic efficacy, providing a promising approach for cancer research [44, 45].

However, it is important to acknowledge certain limitations in this study. Although we observed that the ZIF-8@ICG@Tax+NIR group exhibited the highest cellular uptake, further investigation is needed to elucidate the underlying mechanisms. Nevertheless, based on previous research, we can offer a possible explanation for this phenomenon. Previous reports have indicated that laser hyperthermia can enhance cell membrane permeability and fluidity, thereby promoting the uptake of nanoparticles by cancer cells [36]. This, in turn, enhances the endocytosis of drugs within cancer cells and leads to significant cellular damage. Additionally, ZIF-8 nanoparticles have been reported to facilitate cellular uptake of drugs, and the mechanism behind this may be attributed to the increased permeability of lysosomal membranes by ZIF-8, thereby facilitating the intracellular transport and release of drugs [37].

## Conclusion

The study utilized the ZIF-8@ICG system to encapsulate Tax and construct a theranostic chemo-photothermal NP system. The pH- and NIR- responsive nanoparticles exhibit remarkable efficacy in targeting and aggregating within tumor cells and tissues, effectively exerting tumoricidal effects and demonstrating excellent antitumor and anti-metastatic effects in vivo. These results support the potential of ZIF-8@ICG@Tax as a promising strategy for the treatment of NSCLC. Notably, the effectiveness of the nanomedicine is superior to that of free Tax, highlighting the importance of nanomedicine in clinical management of lung cancer growth and metastasis. This study provides important insights into the development of innovative therapeutic strategies for lung cancer.

## References

[1] Alexander M, Kim SY, Cheng H. Update 2020: management of non-small cell lung cancer. Lung. 2020;198:897–907. 10.1007/s00408-020-00407-5 33175991 10.1007/s00408-020-00407-5 PMC7656891

[2] Wei G, Wang Y, Yang G, Wang Y, Ju R. Recent progress in nanomedicine for enhanced cancer chemotherapy. Theranostics. 2021;11:6370–92. 10.7150/thno.57828 33995663 10.7150/thno.57828 PMC8120226

[3] Duma N, Santana-Davila R, Molina JR. Non-small cell lung cancer: epidemiology, screening, diagnosis, and treatment. Mayo Clin Proc. 2019;94:1623–40. 10.1016/j.mayocp.2019.01.013 31378236 10.1016/j.mayocp.2019.01.013

[4] Bockamp E, Rosigkeit S, Siegl D, Schuppan D. Nano-enhanced cancer immunotherapy: immunology encounters nanotechnology. Cells. 2020;9:2102. 10.3390/cells9092102 32942725 10.3390/cells9092102 PMC7565449

[5] Nie W, Wu G, Zhang J, Huang LL, Ding J, Jiang A, et al. Responsive exosome nano-bioconjugates for synergistic cancer therapy. Angew Chem Int Ed Engl. 2020;59:2018–22. 10.1002/anie.201912524 31746532 10.1002/anie.201912524

[6] Zhu L, Chen L. Progress in research on paclitaxel and tumor immunotherapy. Cell Mol Biol Lett. 2019;24:40. 10.1186/s11658-019-0164-y 31223315 10.1186/s11658-019-0164-y PMC6567594

[7] Sohrabi Kashani A, Packirisamy M. Cancer-nano-interaction: from cellular uptake to mechanobiological responses. Int J Mol Sci. 2021;22:9587. 10.3390/ijms22179587 34502495 10.3390/ijms22179587 PMC8431109

[8] Spitsyna AS, Poryvaev AS, Sannikova NE, Yazikova AA, Kirilyuk IA, Dobrynin SA, et al. Stability of ZIF-8 nanoparticles in most common cell culture media. Molecules. 2022;27:3240 10.3390/molecules27103240 35630717 10.3390/molecules27103240 PMC9144353

[9] Abdelhamid HN. Zeolitic imidazolate frameworks (ZIF-8) for biomedical applications: a review. Curr Med Chem. 2021;28:7023–75. 10.2174/0929867328666210608143703 34102965 10.2174/0929867328666210608143703

[10] Paul A, Banga IK, Muthukumar S, Prasad S. Engineering the ZIF-8 Pore for electrochemical sensor applications—a mini review. ACS Omega. 2022;7:26993–7003. 10.1021/acsomega.2c00737 35967010 10.1021/acsomega.2c00737 PMC9366767

[11] Yu C, Kim YJ, Kim J, Eum K. ZIF-L to ZIF-8 transformation: morphology and structure controls. Nanomaterials. 2022;12:4224. 10.3390/nano12234224 36500846 10.3390/nano12234224 PMC9740542

[12] Ramos VC, Reyes CBG, García GM, Quesada M, Barrero F, Rábago J, et al. ZIF-8 and its magnetic functionalization as vehicle for the transport and release of ciprofloxacin. Pharmaceutics. 2022;14:2546. 10.3390/pharmaceutics14112546 36432737 10.3390/pharmaceutics14112546 PMC9693427

[13] Mi X, Hu M, Dong M, Yang Z, Zhan X, Chang X, et al. Folic acid decorated zeolitic imidazolate framework (ZIF-8) loaded with baicalin as a nano-drug delivery system for breast cancer therapy. Int J Nanomed. 2021;16:8337–52. 10.2147/IJN.S340764 10.2147/IJN.S340764 PMC8714011 34992370

[14] Pomeroy AE, Schmidt EV, Sorger PK, Palmer AC. Drug independence and the curability of cancer by combination chemotherapy. Trends Cancer. 2022;8:915–29. 10.1016/j.trecan.2022.06.009 35842290 10.1016/j.trecan.2022.06.009 PMC9588605

[15] Okusaka T, Furuse J. Recent advances in chemotherapy for pancreatic cancer: evidence from Japan and recommendations in guidelines. J Gastroenterol. 2020;55:369–82. 10.1007/s00535-020-01666-y 31997007 10.1007/s00535-020-01666-y PMC7080663

[16] Davern M, Lysaght J. Cooperation between chemotherapy and immunotherapy in gastroesophageal cancers. Cancer Lett. 2020;495:89–99. 10.1016/j.canlet.2020.09.014 32950619 10.1016/j.canlet.2020.09.014

[17] Jin H, Wang L, Bernards R. Rational combinations of targeted cancer therapies: background, advances and challenges. Nat Rev Drug Discov. 2023;22:213–34. 10.1038/s41573-022-00615-z 36509911 10.1038/s41573-022-00615-z

[18] Zhi D, Yang T, O'Hagan J, Zhang S, Donnelly RF. Photothermal therapy. J Control Release. 2020;325:52–71. 10.1016/j.jconrel.2020.06.032 32619742 10.1016/j.jconrel.2020.06.032

[19] Li X, Lovell JF, Yoon J, Chen X. Clinical development and potential of photothermal and photodynamic therapies for cancer. Nat Rev Clin Oncol. 2020;17:657–74. 10.1038/s41571-020-0410-2 32699309 10.1038/s41571-020-0410-2

[20] Chao AH, Schulz SA, Povoski SP. The application of indocyanine green (ICG) and near-infrared (NIR) fluorescence imaging for assessment of the lymphatic system in reconstructive lymphaticovenular anastomosis surgery. Expert Rev Med Devices. 2021;18:367–74. 10.1080/17434440.2021.1900725 33686906 10.1080/17434440.2021.1900725

[21] Xiong X, Li J, Gao D, Sheng Z, Zheng H, Liu W. Cell-membrane biomimetic indocyanine green liposomes for phototheranostics of echinococcosis. Biosensors. 2022;12:311. 10.3390/bios12050311 35624612 10.3390/bios12050311 PMC9138668

[22] Houthoofd S, Vuylsteke M, Mordon S, Fourneau I. Photodynamic therapy for atherosclerosis. The potential of indocyanine green. Photodiagn Photodyn Ther. 2020;29:101568. 10.1016/j.pdpdt.2019.10.003 10.1016/j.pdpdt.2019.10.003 31627015

[23] Lau CT, Au DM, Wong KKY. Application of indocyanine green in pediatric surgery. Pediatr Surg Int. 2019;35:1035–41. 10.1007/s00383-019-04502-4 31243546 10.1007/s00383-019-04502-4

[24] Fu S, Li G, Zang W, Zhou X, Shi K, Zhai Y. Pure drug nano-assemblies: a facile carrier-free nanoplatform for efficient cancer therapy. Acta Pharm Sin B. 2022;12:92–106. 10.1016/j.apsb.2021.08.012 35127374 10.1016/j.apsb.2021.08.012 PMC8799886

[25] Egloff-Juras C, Bezdetnaya L, Dolivet G, Lassalle HP. NIR fluorescence-guided tumor surgery: new strategies for the use of indocyanine green. Int J Nanomed. 2019;14:7823–38. 10.2147/IJN.S207486 10.2147/IJN.S207486 PMC6768149 31576126

[26] Wang T, Li S, Zou Z, Hai L, Yang X, Jia X, et al. A zeolitic imidazolate framework-8-based indocyanine green theranostic agent for infrared fluorescence imaging and photothermal therapy. J Mater Chem B. 2018;6:3914–21. 10.1039/c8tb00351c 32254319 10.1039/c8tb00351c

[27] Xi D, Xiao M, Cao J, Zhao L, Xu N, Long S, et al. NIR light-driving barrier-free group rotation in nanoparticles with an 88.3% photothermal conversion efficiency for photothermal therapy. Adv Mater. 2020;32:e1907855. 10.1002/adma.201907855 32022978 10.1002/adma.201907855

[28] Tian G, Wu Y, Jin X, Zeng Z, Gu X, Li T, et al. The incidence rate and influence factors of hemolysis, lipemia, icterus in fasting serum biochemistry specimens. PLoS One. 2022;17:e0262748. 10.1371/journal.pone.0262748 35045128 10.1371/journal.pone.0262748 PMC8769349

[29] Wang Q, Sun Y, Li S, Zhang P, Yao Q. Synthesis and modification of ZIF-8 and its application in drug delivery and tumor therapy. RSC Adv. 2020;10:37600–20. 10.1039/d0ra07950b 35515141 10.1039/d0ra07950b PMC9057214

[30] Shi Z, Chen X, Zhang L, Ding S, Wang X, Lei Q, et al. FA-PEG decorated MOF nanoparticles as a targeted drug delivery system for controlled release of an autophagy inhibitor. Biomater Sci. 2018;6:2582–90. 10.1039/c8bm00625c 30151542 10.1039/c8bm00625c

[31] Luzuriaga MA, Benjamin CE, Gaertner MW, Lee H, Herbert FC, Mallick S, et al. ZIF-8 degrades in cell media, serum, and some-but not all-common laboratory buffers. Supramol Chem. 2019;31:485–90. 10.1080/10610278.2019.1616089 31892768 10.1080/10610278.2019.1616089 PMC6938391

[32] Yang Y, Liu J, Liang C, Feng L, Fu T, Dong Z, et al. Nanoscale metal-organic particles with rapid clearance for magnetic resonance imaging-guided photothermal therapy. ACS Nano. 2016;10:2774–81. 10.1021/acsnano.5b07882 26799993 10.1021/acsnano.5b07882

[33] Wan M, Lv S, Hong T, Zhao Y, Peng L, Sun L. Carboxymethyl β-cyclodextrin grafted hollow copper sulfide@mesoporous silica carriers for stimuli-responsive pesticide delivery. Colloids Surf B Biointerfaces. 2023;228:113425. 10.1016/j.colsurfb.2023.113425 37384965 10.1016/j.colsurfb.2023.113425

[34] Zhu D, Lu Y, Gui L, Wang W, Hu X, Chen S, et al. Self-assembling, pH-responsive nanoflowers for inhibiting PAD4 and neutrophil extracellular trap formation and improving the tumor immune microenvironment. Acta Pharm Sin B. 2022;12:2592–608. 10.1016/j.apsb.2021.11.006 35646534 10.1016/j.apsb.2021.11.006 PMC9136569

[35] Ando H, Ikeda A, Tagami M, Matsuo N, Shimizu T, Ishima Y, et al. Oral administration of sodium bicarbonate can enhance the therapeutic outcome of Doxil® via neutralizing the acidic tumor microenvironment. J Control Release. 2022;350:414–20. 10.1016/j.jconrel.2022.08.031 35988781 10.1016/j.jconrel.2022.08.031

[36] Chen X, Fan X, Zhang Y, Wei Y, Zheng H, Bao D, et al. Cooperative coordination-mediated multi-component self-assembly of “all-in-one” nanospike theranostic nano-platform for MRI-guided synergistic therapy against breast cancer. Acta Pharm Sin B. 2022;12:3710–25. 10.1016/j.apsb.2022.02.027 36176903 10.1016/j.apsb.2022.02.027 PMC9513557

[37] Zhang S, Li J, Yan L, You Y, Zhao F, Cheng J, et al. Zeolitic imidazolate framework-8 (ZIF-8) as a drug delivery vehicle for the transport and release of telomerase inhibitor BIBR 1532. Nanomaterials. 2023;13:1779. 10.3390/nano13111779 37299682 10.3390/nano13111779 PMC10254680

[38] Lin ID, Lin K-H. The impact of adverse drug reactions on medication adherence and outpatient treatment outcomes in female breast cancer: a review protocol. J Adv Nurs. 2023;79:825–31. 10.1111/jan.15542 36524324 10.1111/jan.15542

[39] Liu P, Huang Y, Zhan C, Zhang F, Deng C, Jia Y, et al. Tumor-overexpressed enzyme responsive amphiphiles small molecular self-assembly nano-prodrug for the chemo-phototherapy against non-small-cell lung cancer. Mater Today Biol. 2023;21:100722. 10.1016/j.mtbio.2023.100722 10.1016/j.mtbio.2023.100722 PMC10401344 37545562

[40] Lee H, Park H, Noh GJ, Lee ES. pH-responsive hyaluronate-anchored extracellular vesicles to promote tumor-targeted drug delivery. Carbohydr Polym. 2018;202:323–33. 10.1016/j.carbpol.2018.08.141 30287007 10.1016/j.carbpol.2018.08.141

[41] Zhang DY, Liu H, Younis MR, Lei S, Chen Y, Huang P, et al. In-situ TiO(2-x) decoration of titanium carbide MXene for photo/sono-responsive antitumor theranostics. J Nanobiotechnol. 2022;20:53. 10.1186/s12951-022-01253-8 10.1186/s12951-022-01253-8 PMC8796495 35090484

[42] Yang T, Tang Y, Liu L, Lv X, Wang Q, Ke H, et al. Size-dependent Ag(2)S nanodots for second near-infrared fluorescence/photoacoustics imaging and simultaneous photothermal therapy. ACS Nano. 2017;11:1848–57. 10.1021/acsnano.6b07866 28117993 10.1021/acsnano.6b07866

[43] Rizwan M, Roy VAL, Abbasi R, Irfan S, Khalid W, Atif M, et al. Novel 2D MXene cobalt ferrite (CoF@Ti(3)C(2)) composite: a promising photothermal anticancer in vitro study. ACS Biomater Sci Eng. 2024;10:2074–87. 10.1021/acsbiomaterials.3c01328 38111288 10.1021/acsbiomaterials.3c01328

[44] Adepu S, Ramakrishna S. Controlled drug delivery systems: current status and future directions. Molecules. 2021;26:5905. 10.3390/molecules26195905 34641447 10.3390/molecules26195905 PMC8512302

[45] Liu S, Shen C, Jiang D, Qian C, Yang Z, Wang J, et al. Cascade tumor therapy platform for sensitized chemotherapy and penetration enhanced photothermal therapy. Macromol Biosci. 2022;22:e2100429. 10.1002/mabi.202100429 34910842 10.1002/mabi.202100429

